# Study on relationship between bacterial diversity and quality of Huangjiu (Chinese Rice Wine) fermentation

**DOI:** 10.1002/fsn3.2369

**Published:** 2021-06-04

**Authors:** Guangfa Xie, Huajun Zheng, Zheling Qiu, Zichen Lin, Qi Peng, Girma Dula Bealu, Nabil Ibrahim Elsheery, Yin Lu, Chi Shen, Jianwei Fu, Huanyi Yang, Jiongping Han, Jian Lu, Guanming Liu

**Affiliations:** ^1^ College of Biology and Environmental Engineering College of Shaoxing CRW Zhejiang Shuren University Hangzhou China; ^2^ School of Life Science National Engineering Research Center for Chinese CRW (Branch Center) Shaoxing University Shaoxing China; ^3^ Shaoxing Jianhu Brewing Co., Ltd Shaoxing China; ^4^ California Institute of Food and Agricultural Research University of California Davis CA USA; ^5^ Agriculture Botany Department Faculty of Agriculture Tanta University Tanta Egypt; ^6^ School of Medicine Shaoxing University Shaoxing China; ^7^ School of Biotechnology National Engineering Laboratory for Cereal Fermentation Technology Jiangnan University Wuxi China

**Keywords:** gene prediction, Huangjiu, lactic acid bacteria, metagenomic sequencing, spoilage

## Abstract

Huangjiu (Chinese rice wine) is brewed in an open environment, where bacteria play an important role during the fermentation process. In this study, bacterial community structure and composition changes in the fermented mash liquid of mechanized Huangjiu, well‐fermented manual Huangjiu (wines of good qualities), and poorly fermented manual Huangjiu (wines of poor qualities: spoilage, high acidity, low alcohol content) in different fermentation stages from Guyuelongshan Shaoxing Huangjiu company were analyzed via metagenomic sequencing. And bacterial metabolic difference was analyzed via gene prediction of metabolic pathway enzymes. The results showed that the bacterial diversity degree was abundant, and the number of bacterial species in every sample was approximately 200–400. Lactic acid bacteria (LAB) dominated the bacterial community of Huangjiu fermentation, and *lactobacillus* was predominant species in well‐fermented Huangjiu while *Lactobacillus brevis* had an absolute dominance in spoilage Huangjiu. Further, gene prediction revealed that transformation of malate to pyruvate and lactate anabolism was more active in mash liquid of well‐fermented manual Huangjiu, while acetate accumulation was stronger in mash liquid of poorly fermented manual Huangjiu, which explained acidity excess reason in poorly fermented Huangjiu at gene level.

## Graphical abstract

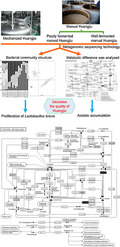

## 1 INTRODUCTION

Huangjiu (Chinese rice wine), a traditional Chinese alcohol beverage, is popular among Chinese customers. Huangjiu is honored as national banquet alcohol beverage due to abundant nutrition and health function (Yongmei et al., 2007; Zhao et al., 2018). Huangjiu is brewed in an open environment, with glutinous rice as raw material and wheat Qu as sacchariferous agent. There are two distinct types of Huangjiu starter culture. One is made of steamed wheat and inoculated pure *A. oryzae*. Another is made of raw wheat and naturally inoculated microorganisms. Mixtures of these starter cultures are used in the modern brewing process of Huangjiu (Cao et al., 2010; Guan et al., 2012; Zhang et al., 2012). It is widely recognized that Huangjiu fermentation is in essence the process of mixed fermentation of yeast, bacteria, and molds to produce alcohol and other metabolites, thus formation of unique flavor and aroma (Chen et al., 2018). Recently, polymerase chain reaction‐denaturation gel electrophoresis (PCR‐DGGE) was employed for analysis of microbial community structure of wheat Qu and fermented mash liquid of Huangjiu (Guan et al., 2012; Yu et al., 2018). However, some disadvantages existed in PCR‐DGGE method, such as preferential DNA amplification of some templates, and the presence of faint bands on gel images that may be difficult to be seen by naked eye, which resulted in underestimation of microbial diversity in the Huangjiu samples (Garofalo et al., 2017; Milanovic et al., 2017). With the advance of high‐throughput sequencing, metagenomic sequencing detecting microbial diversity in the whole environmental system has been increasingly mature. Because of huge data volume, ability to detect all microbes including uncultured ones, metagenomic sequencing has been extensively used in fecal and food microbiota analysis (Chen et al., 2020; Lyu et al., 2021; Mao et al., 2015; Xie et al., 2013). For example, Liu et al. (Liu et al., 2018) identified *Burkholderia* and *Bacillus*as predominant bacterial genus in Gu Tian Qu (71.62%) and Wu Yi Hong Qu (44.73%), respectively, and detected 213 bacterial genus with low abundance via Illumina‐based metagenomic sequencing.

**FIGURE 1:**
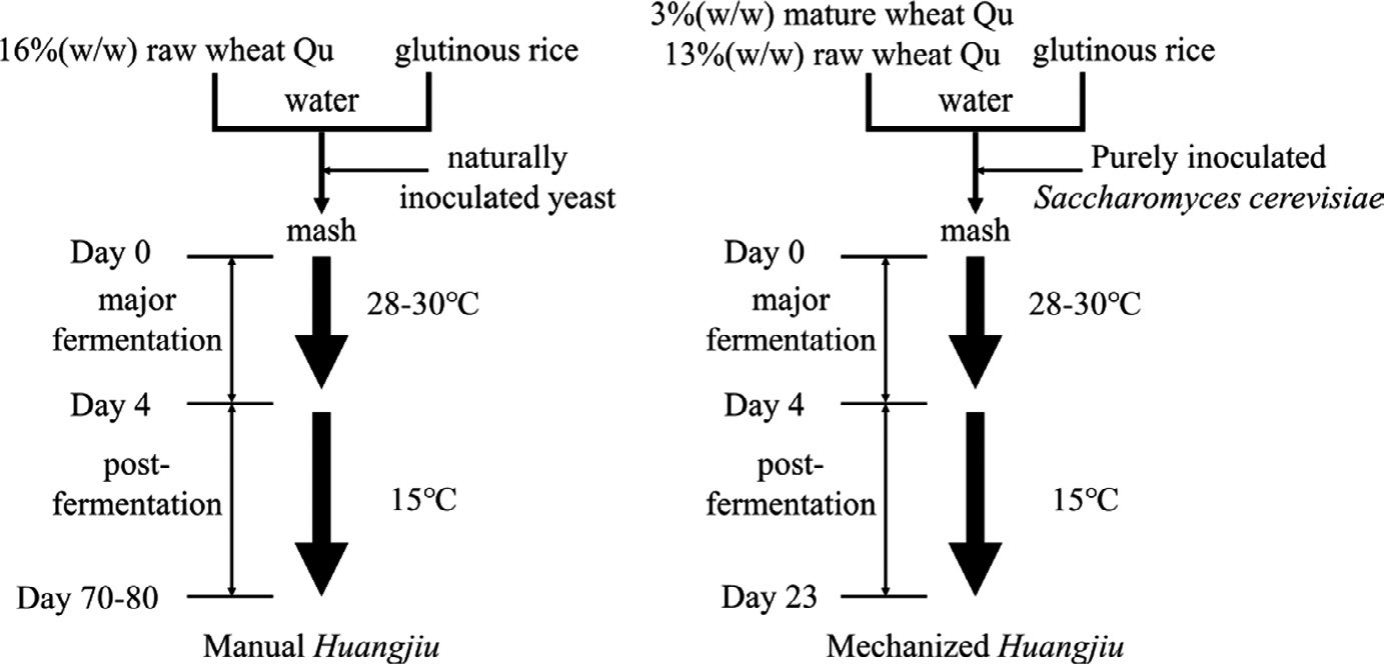
The scheme of manual and mechanized Huangjiu fermentation. (raw wheat Qu contains naturally inoculated mildew and mature wheat Qu contains purely inoculated Aspergillus oryzae, which are employed to hydrolyze raw material)

The brewing technology of Huangjiu is divided into manual Huangjiu and mechanized Huangjiu (Figure 1). The mechanized Huangjiu is fermented in a large stainless steel tank with yeast and wheat starter as saccharifying and fermenting agents. The distiller's yeast is cultivated by pure breed, and the wheat koji is mixed by natural culture and pure breed. Traditional manual Huangjiu uses naturally cultivated wheat koji and yeast as saccharifying and fermenting agents. In the first fermentation stage, pottery jar is used as fermentation container, and in the second fermentation stage, pottery jar is used as fermentation container. Poor quality sacchariferous agents, inappropriate ratio of raw material and unsuitable control of fermentation temperature may lead to poorly fermented Huangjiu (Total acid is over standard, alcohol content is below standard). At the end of fermentation, the control standard for Huangjiu fermentation mash by Shaoxing Huangjiu production enterprises is as follows: the alcohol content is above 18%vol and the total acid is below 6.5 g/L.

Although little literature was available on microbial composition in spoilage Huangjiu, beer spoilage was mainly due to LAB (Geissler et al., 2016; Schneiderbanger et al., 2018). As bacteria was important in Huangjiu brewing, this study aimed at analyses bacterial community diversity of fermented mash liquid of mechanized Huangjiu, well‐fermented manual Huangjiu and poorly fermented manual Huangjiu, and figuring out the explanation for spoilage in poorly fermented manual Huangjiu. The results would provide practical guidance for quality control of Huangjiu industry.

## 2 MATERIALS AND METHODS

### 2.1 Sample

The fermented mash of Huangjiu was obtained from Guyuelongshan Shaoxing Huangjiu Company, including the fermented mash liquid of mechanized Huangjiu, well‐fermented manual Huangjiu, and poorly fermented manual Huangjiu (spoilage). Samples were stored at −80℃ before genome DNA extraction. For manual Huangjiu with unstable fermentation, we collected 24 batches of samples at different fermentation stages from 6 batches of samples in 4 workshops during the brewing season. Two representative batches of samples were selected for Macro‐sequencing analysis. For mechanized rice wine, due to its stable quality, we only took a batch of samples from normal fermentation for Macro‐sequencing analysis. Therefore, the samples have good representativeness. Three replicates were performed.

### 2.2 Physicochemical indexes determination

Alcohol content and acidity of Huangjiu samples were determined according to the protocol recommended by national standard of Chinese Huangjiu GB/T 13662‐2018 (Standardization administration, 2018).

### 2.3 DNA extraction

MP Fast DNA SPIN Kit for Soil kit (MP Biomedical) was used to extract genomic DNA from the sample. The extracted DNA was determined by NanoDrop nucleic acid quantitative assay. DNA was re‐extracted from the sample with concentration less than 5 ng/μl. The extracted DNA was stored at −20℃ for metagenomic sequencing. Three replicates were performed.

### 2.4 Metagenomic sequencing

The metagenomic DNA libraries were constructed with 2 μg genome DNA according to the IlluminaTruSeq DNA Sample Prep v2 Guide, with an average of 350 bp insert size (Qin et al., 2014). The quality of DNA libraries was evaluated via Agilent bioanalyzer with a DNA LabChip 1000 kit. Sequencing was carried out in Illumina Hiseq2500. Three replicates were performed.

#### 2.4.1 Meatgenomic data analysis

High‐quality data after sequencing quality control was aligned to microbial genomes available on NCBI (National Center for Biotechnology Information) and Gen bank, thus identifying the microbial species and calculating its abundance (N. Qin et al., 2014). Three replicates were performed.

#### 2.4.2 De novo assembly of illumina high‐quality reads

De bruijn‐graph‐based assembler SOAPdenovo was utilized to assemble short reads with parameter k‐mers, varying from 39 to 59. N50 was calculated for contigs of different k‐mers and only the contigs of largest N50 assembly were attributed to a sample (Qin et al., 2014). In order to utilize as many reads as possible, the unassembled reads from the same batch of fermentation were merged for a second assembly. All these contigs were used for gene prediction.

#### 2.4.3 Gene prediction and contig origin determination

To avoid rice or wheat gene pollution, SOAPdenovo assembled contigs were aligned to cereal reference genome. Contigs with coverage greater than 90% and similarity greater than 95% were considered to be contaminated and removed. The remaining contig was mapped to bacterial genome database and subject to gene prediction via MetaGeneMark software. Non‐redundant gene encoding proteins were aligned to KEGG database, and a protein was assigned if E value was smaller than 1 × e^−10^, therefore its gene function could be predicted (Qin et al., 2012).

## 3 RESULTS AND DISCUSSION

### 3.1 Physicochemical indexes analysis of different fermented mash liquid of Huangjiu

Alcohol content and acidity of different Huangjiu fermentation mash were described in Table 1. As wheat Qu (It is made of steamed wheat and inoculated pure *A. oryzae*) was used to accelerate saccharification of glutinous rice in mechanized Huangjiu. The saccharification fermentation speed of mechanized Huangjiu is faster, the alcohol content could usually reach 13%vol on the fourth day (Zhang et al., 2012). However, traditional manual Huangjiu fermented relatively slowly, and the alcohol content reached 13%vol on the seventh day. The acidity of Huangjiu should be controlled below 6.5 g/L to maintain good quality. The results showed that acidity of both mechanized Huangjiu and well‐fermented manual Huangjiu reached the standard, and spoilage Huangjiu reached the acidity standard at the end of major fermentation (14d), but the acidity elevated obviously and exceeded standard in the postfermentation.

**TABLE 1:** The physiochemical indexes of fermented mash liquid of mechanized Huangjiu, well‐fermented manual Huangjiu, and poorly fermented manual Huangjiu (spoilage, high acidity, low alcohol) samples

Sample	Sampling time (d)	Alcohol degree (%vol)	Acidity (g L^−1^)
Mechanized Huangjiu^a^ (machine)			
Machine_2	2 days	12.4 ± 0.9^efg^	4.5 ± 0.6^c^
Machine_4	4 days	13.5 ± 1.4^cdefg^	4.7 ± 0.4^c^
Machine_7	7 days	15.3 ± 1.4^bcde^	4.8 ± 1.0^c^
Machine_14	14 days	17.1 ± 1.9^abc^	5.0 ± 0.8^bc^
Machine_16	16 days	18.2 ± 1.9^ab^	5.4 ± 1.0^bc^
Spoilage Huangjiu^b^ (poor manual)			
Poor manual_2	2 days	10.2 ± 1.0^g^	5.8 ± 0.4^abc^
Poor manual_4	4 days	11.5 ± 1.0^fg^	6.5 ± 0.9^abc^
Poor manual_7	7 days	12.7 ± 1.1^defg^	6.4 ± 0.5^abc^
Poor manual_14	14 days	14.2 ± 1.0^cdef^	6.5 ± 0.6^abc^
Poor manual_30	30 days	15.6 ± 0.8^bcde^	7.2 ± 0.9^ab^
Poor manual_60	60 days	18.1 ± 0.9^ab^	8.0 ± 0.7^a^
Well‐fermented Huangjiu^b^ (good manual)			
Good manual_2	2 days	10.3 ± 1.0^g^	5.8 ± 1.1^abc^
Good manual_4	4 days	11.3 ± 0.9^fg^	5.9 ± 0.9^abc^
Good manual_7	7 days	13.5 ± 0.9^cdefg^	5.8 ± 0.7^abc^
Good manual_14	14 days	14.3 ± 0.7^cdef^	6.0 ± 1.3^abc^
Good manual_30	30 days	16.2 ± 1.7^abcd^	6.2 ± 0.8^abc^
Good manual_60	60 days	19.5 ± 1.1^a^	6.1 ± 0.4^abc^

Note

a. The fermentation of mechanized Huangjiu lasts for about 20 days.

b. The fermentation of manual Huangjiu lasts for about 60 days.

c. Values are means of triplicates ± *SD*.

d. Values are significantly different (*p* < .05) labeled by different superscript letters within a column.

### 3.2 Bacterial diversity of different fermented mash liquid of Huangjiu

**FIGURE 2:**
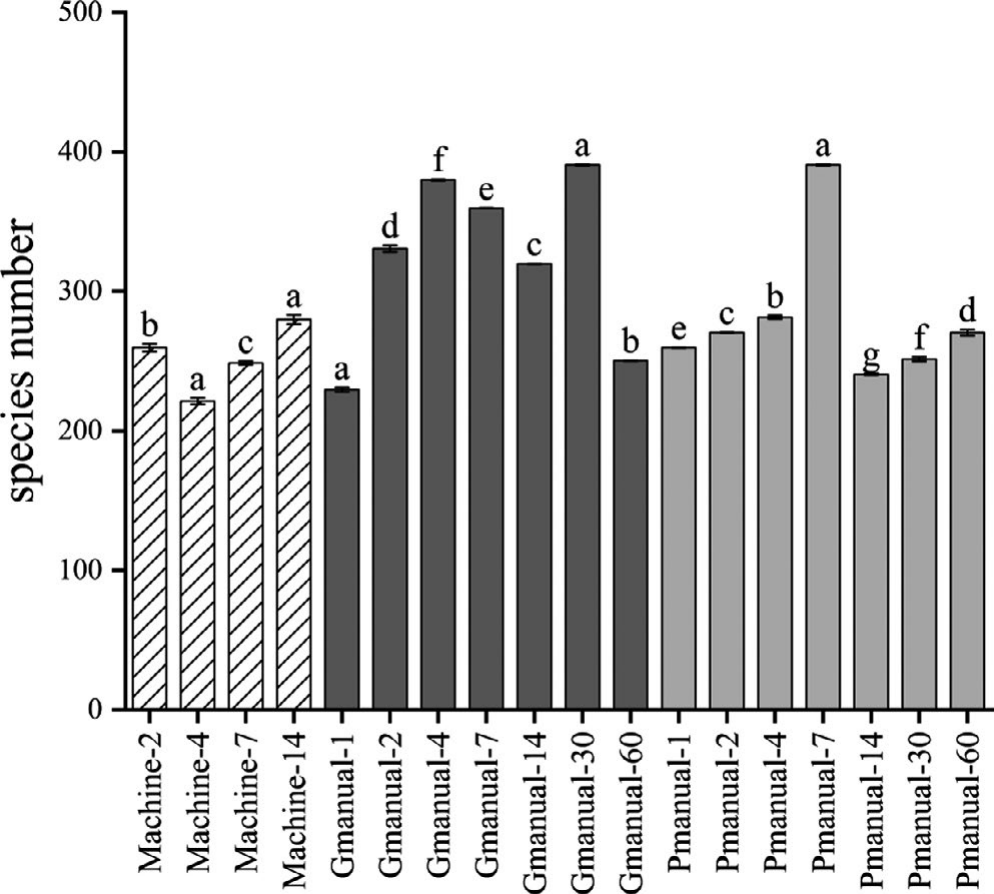
Bacterial species number of fermented mash liquid of mechanized Huangjiu, well‐fermented manual Huangjiu and poorly fermented manual Huangjiu (spoilage, high acidity, low alcohol) samples during fermentation. (machine n: mechanized Huangjiu; good manual n: well‐fermented manual Huangjiu; poor manual n: spoilage manual Huangjiu. The word “n” represents the day of sampling)

**FIGURE 3:**
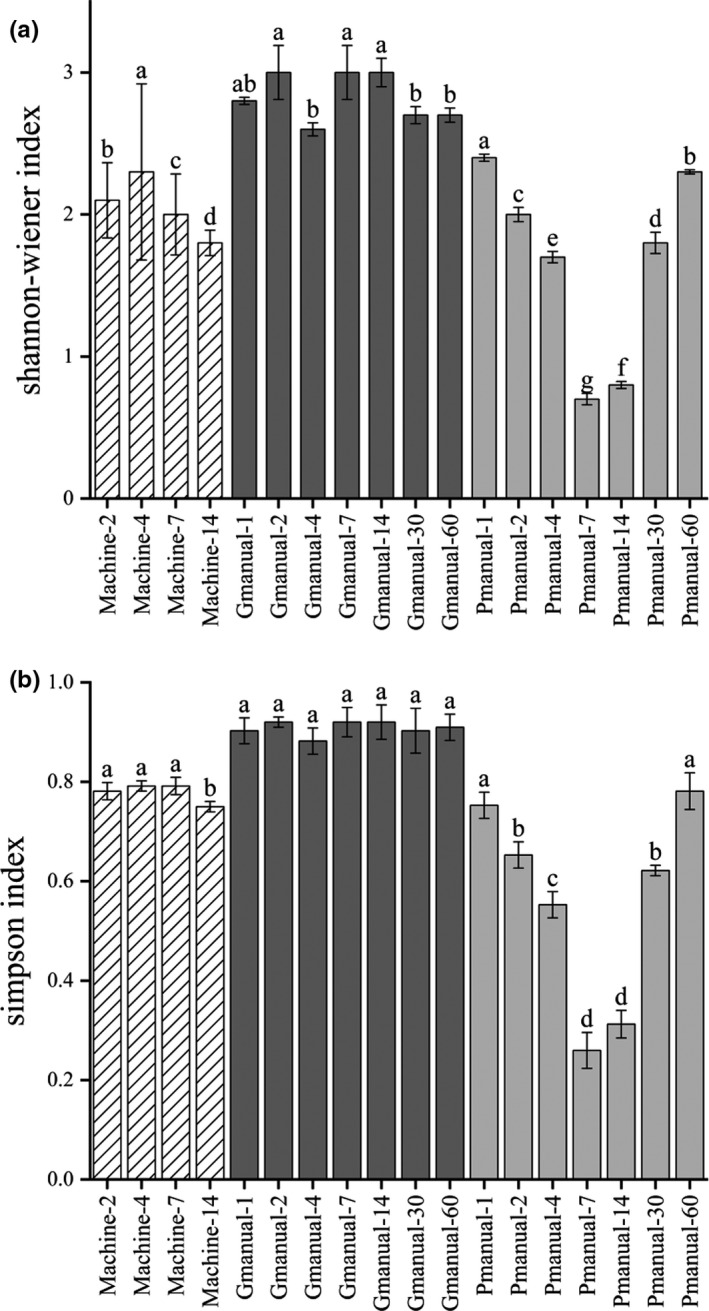
Bacterial diversity indexes (Shannon‐wiener index and Simpson index) of fermented mash liquid of mechanized Huangjiu, well‐fermented manual Huangjiu and poorly fermented manual Huangjiu (spoilage, high acidity, low alcohol) samples during fermentation

Huangjiu fermentation tank is open to air. 13% naturally cultured raw wheat Qu is used in the mechanized Huangjiu fermentation process, 16% naturally cultured raw wheat Qu is for manual Huangjiu. It contains a large number of microorganisms. Various microbes interact to produce specific flavor and aroma compounds, and even mechanized Huangjiu fermentation is also open. Bacterial diversity analyzed via metagenomic sequencing was shown in Figure 2. During the fermentation process of three different fermented mash liquid of Huangjiu, bacterial diversity level was high, with bacterial species number ranging from 200 to 400. Well‐fermented manual Huangjiu had the most abundant bacterial species, while bacterial species number of mechanized Huangjiu was relatively lower than manual one. This may be the result of pure *S. cerevisiae* and *A. oryzae* that speeded up alcohol fermentation to inhibit growth of a part of bacteria to some extent. At the same time, compared with manual Huangjiu, fewer bacteria were brought into the production of mechanized Huangjiu using saccharification starter. Shannon‐wiener and Simpson index of three different fermented mash liquid of Huangjiu were revealed in Figure 3. These two indexes were indicative of microbial diversity, and the higher the indexes were, the more abundant diversity was (Valverde et al., 2016). The results revealed that well‐fermented manual Huangjiu samples had the highest bacterial diversity. In addition, diversity changes in mechanized Huangjiu and well‐fermented manual Huangjiu samples remained relatively stable, while the variation of bacterial diversity was significantly fluctuated in spoilage manual Huangjiu and the diversity degree of this sample was the lowest on seventh day.

### 3.3 Bacterial composition analysis

**FIGURE 4:**
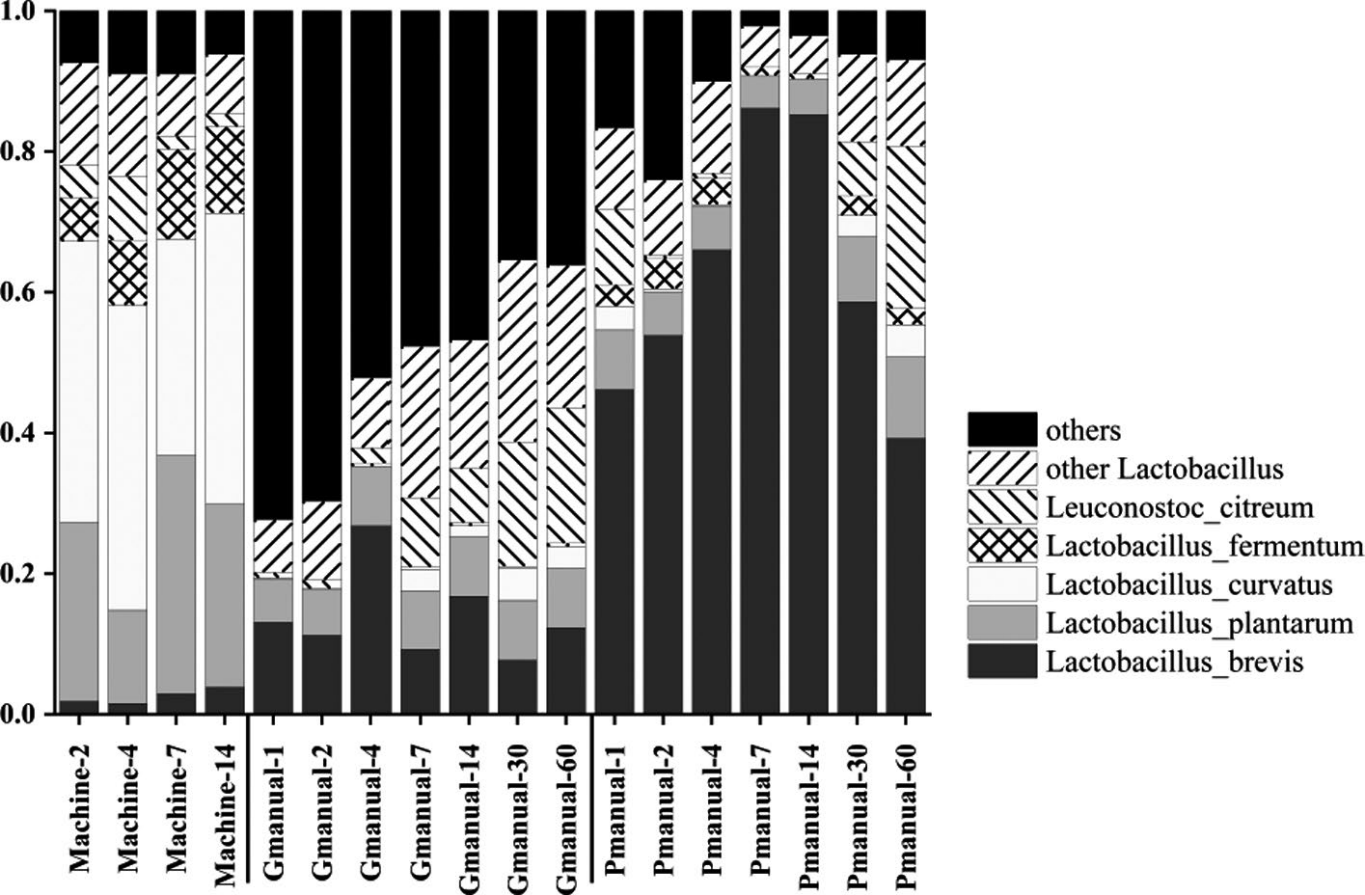
Bacterial composition of fermented mash liquid of mechanized Huangjiu, well‐fermented manual Huangjiu and poorly fermented manual Huangjiu (spoilage, high acidity, low alcohol) samples during fermentation

**FIGURE 5:**
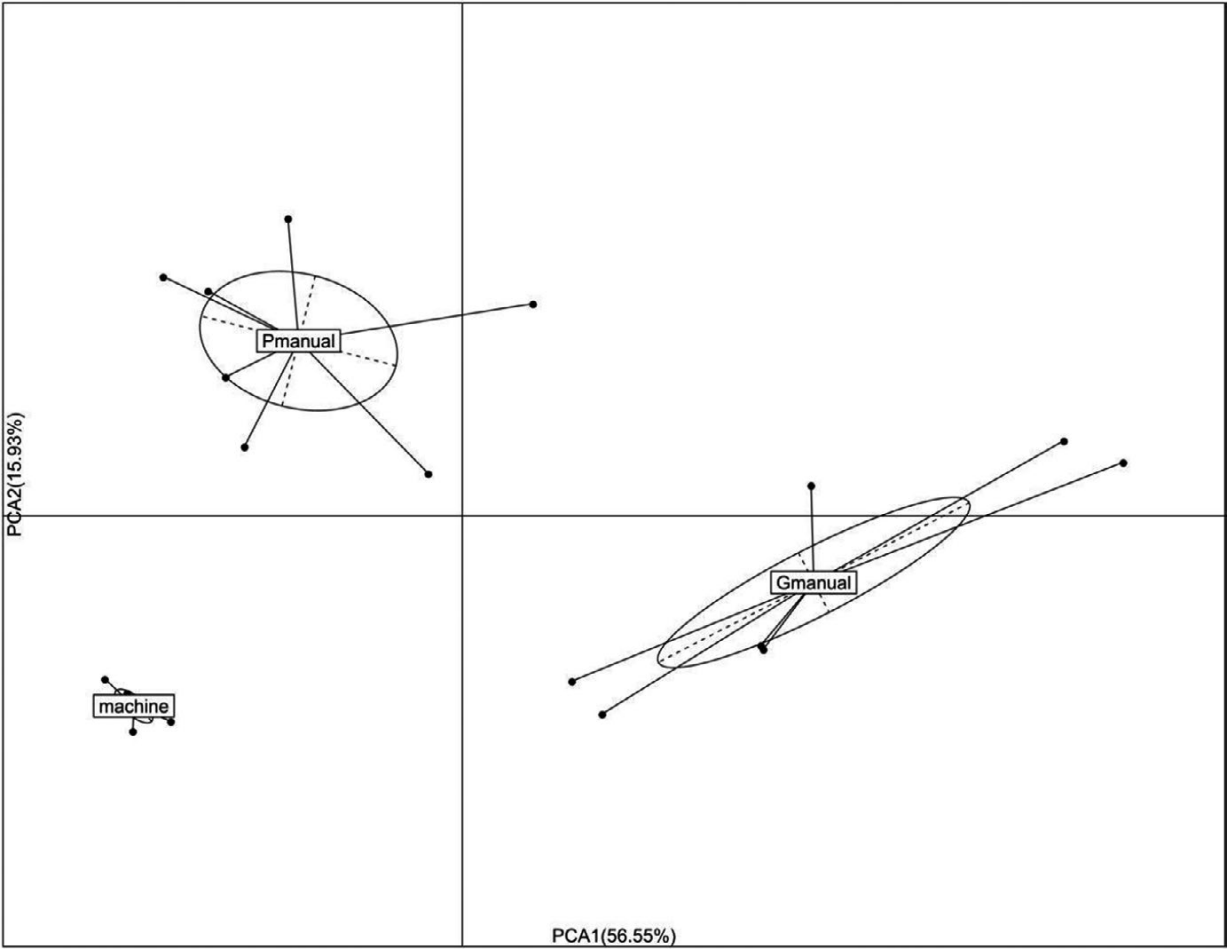
PCA analysis of fermented mash liquid of mechanized Huangjiu, well‐fermented manual Huangjiu and poorly fermented manual Huangjiu (spoilage, high acidity, low alcohol) samples based on bacterial species contribution to the bacterial community structure

Bacterial composition detected via Illumina‐based metagenomic sequencing was shown in Figure 4. LAB, especially lactobacillus dominated the bacterial community of different fermented mash liquid of Huangjiu samples, suggesting that LAB was the predominant bacterial species in Huangjiu fermentation, which was consistent with the PCR‐DGGE results (Yu et al., 2018). However, PCR‐DGGE could hardly detect bacteria species with very low abundance, while 121 lactic acid bacteria were detected via high‐throughput sequencing in the present study (Table 2), providing more detailed information of LAB in Huangjiu fermentation. Bacterial species except LAB were classified into “others,” among which *Saccharopolyspora*, *Bacillus*, *Pantoea* possessed higher proportion. As LAB has strong acid‐producing capacity, proper proliferation of them in Huangjiu fermentation reduces pH value of fermentation mash and therefore inhibits growth of other infectious microbes. PCA analysis based on LAB species contribution showed that there was little difference among the mechanized Huangjiu samples, but there was a significant difference among the well‐fermented manual Huangjiu samples (including spoilage Huangjiu) (Figure 5), which indicated that the quality of the mechanized Huangjiu was more easily controlled.

**TABLE 2:** Species number of LAB genera detected in Huangjiu samples

LAB genera	Species number
*Lactobacillus*	50
*Lactococcus*	2
*Leuconostoc*	11
*Weissella*	4
*Pediococcus*	4
*Oneococcus*	2
*Tetragenococcus*	1
*Bifidobactrium*	6
*Atopobium*	1
*Carnobacterium*	2
*Granulicatella*	1
*Enterococcus*	10
*Streptococcus*	27

In the mechanized fermented liqueur mash, *Lactobacillus plantarum* accounted for a large proportion, while the *L. plantarum* could secrete bacteriocins with antibacterial activity, which gave it an advantage in fermentation (Navarro et al., 2000). The proportion of LAB was particularly high in fermented mash liquid of spoilage Huangjiu, and *Lactobacillus brevis* was the dominant bacterium. Studies have shown that *L. brevis* can lead to the rancidity of beer and grape wine and can produce unpleasant odors and pungent flavor substances (Costello et al., 2001). In fermented mash liquid of spoilage Huangjiu, *L. brevis* dominated the composition of bacteria from the fourth day of fermentation, reaching the highest proportion on the seventh day. The growth of other bacteria was inhibited by *L. brevis*, which destroyed the synergistic effect of multiple strains. However, this was the most critical period for the fermentation of manual Huangjiu. Due to the strong acid production capacity of *L. brevis*, the excessive growth led to the rancidity of Huangjiu, which had a great impact on the quality of Huangjiu. Therefore, the quantity of *L. brevis* during the fermentation should be listed as one of the objects to monitor the fermentation process in order to take timely measures to reduce the loss of spoilage.

### 3.4 Gene function prediction

**FIGURE 6:**
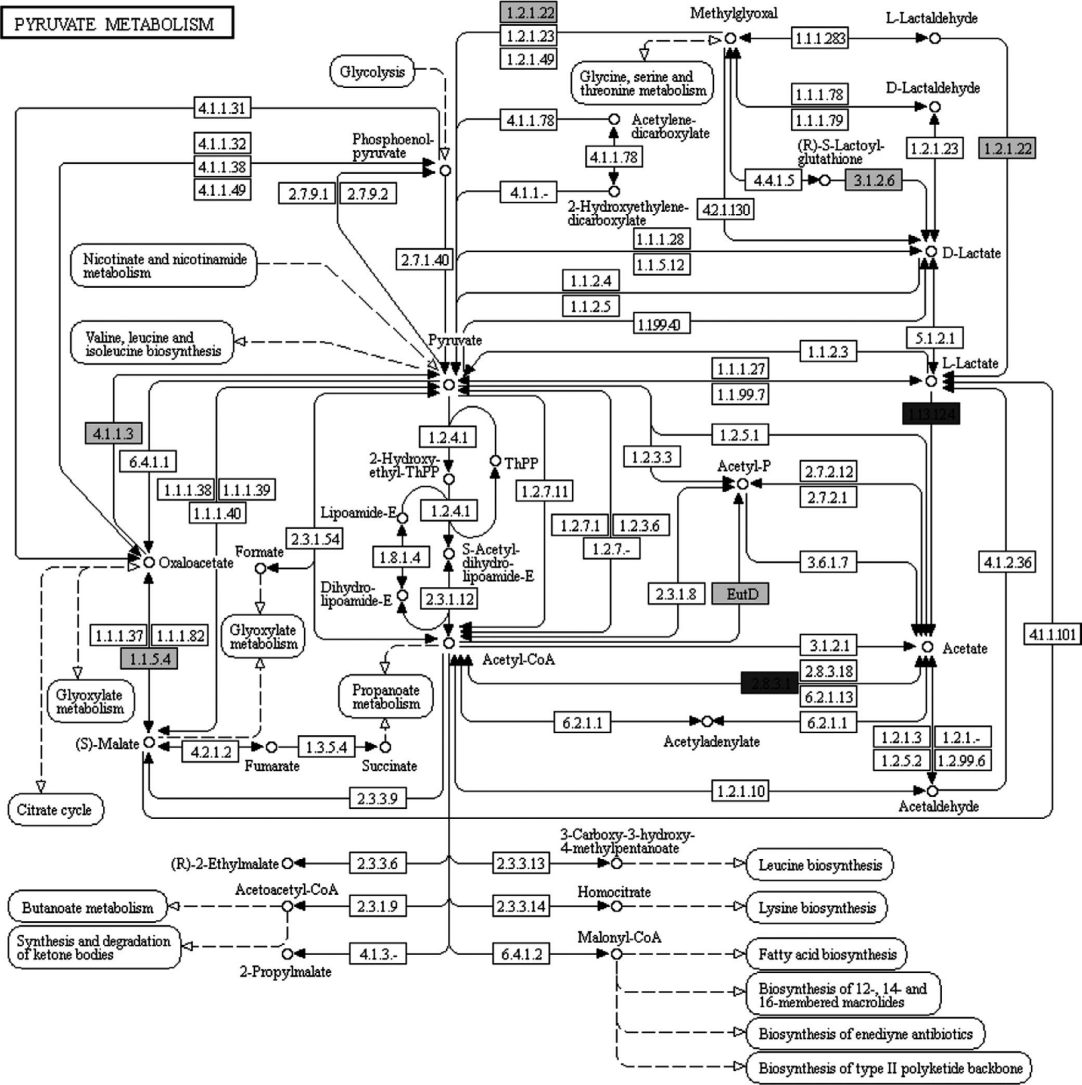
Metabolic network of bacterial community in Huangjiu fermentation. The gray and black rectangle represent enzyme encoding by higher abundance of gene in fermented mash liquid of well‐fermented manual Huangjiu and poorly fermented manual Huangjiu (spoilage, high acidity, low alcohol), respectively (difference above 5 times)

Metagenomic sequencing has an obvious advantage, that is, enormous sequence data size. Not only can researchers calculate the relative abundance of species in microbial community, but predict relevant gene function according to sequence alignment with gene sequence in known genome. Figure 6 described the metabolic network in the microbial cells, where number in rectangle represented enzyme (Table 3) catalyzing the corresponding biochemical reactions. The results showed that genes coding for malate‐pyruvate bioconversion (1.1.5.4 and 4.1.1.3) were more abundant in well‐fermented Huangjiu, thus reducing malate accumulation and improving Huangjiu quality, a feature which was similar to malolactic fermentation in wine (Neto et al., 2016). In addition, lactate anabolisms (1.2.1.22 and 3.1.2.6) were also more active in well‐fermented Huangjiu than that in spoilage Huangjiu. However, it was worth noticing that acetate accumulation (1.13.12.4 and 2.8.3.1) genes were richer in spoilage Huangjiu. From the above mentioned, it could be concluded that excessive accumulation of acetate rather than lactate was the main reason of spoilage in Huangjiu. The present study demonstrated that metagenomic sequencing could be employed to explain some phenomenon in food industry at gene level.

**TABLE 3:** EC number and its corresponding enzyme shown in metabolic network with above 5 times difference between well‐fermented and spoilage Huangjiu samples

EC number	Enzyme
1.2.1.22	lactaldehyde reductase
3.1.2.6	Hydro acyl glutathione hydrolase
4.1.1.3	oxaloacetate decarboxylase subunit gamma
1.1.5.4	malate dehydrogenase
Eut D	phosphate acetyltransferase
1.13.12.4	lactate oxidase
2.8.3.1	acyl CoA: acetate CoA transferase

## 4 CONCLUSION

In this study, metagenomic sequencing was employed to analyze bacterial diversity in fermented mash liquid of mechanized Huangjiu, well‐fermented manual Huangjiu, and poorly fermented manual Huangjiu (spoilage, high acidity, low alcohol content). The results showed that every sample has 200–400 bacteria species, with LAB dominating the bacterial community. However, excessive proliferation of *Lactobacillus brevis* was observed in spoilage Huangjiu. Gene prediction proved that lactate and acetate accumulation was stronger in fermented mash liquid of well‐fermented manual Huangjiu and poorly fermented manual Huangjiu (spoilage), respectively, which revealed that acetate was the main reason of Huangjiu spoilage. Gene prediction analysis showed that metabolic genes such as malate–pyruvate and biotin biosynthesis were expressed more in fermented mash liquid of well‐fermented manual Huangjiu than those in fermented mash liquid of poorly fermented manual Huangjiu (spoilage, high acidity, low alcohol content). Metagenomic sequencing technology was applied to the analysis of bacterial community structure in the process of Huangjiu fermentation, which made up for the deficiency of isolation culture and PCR‐DGGE methods in the analysis of bacterial diversity, providing an effective means to further understand the role of bacteria, especially *Lactobacillus* in Huangjiu fermentation.

## CONFLICTS OF INTEREST

There are no conflicts of interest in this manuscript.

## ETHICAL APPROVAL

This study does not involve any human or animal testing.

## DECLARATIONS

Compliance with ethical standards.

## CODE AVAILABILITY

Software applications are available.

## INFORMED CONSENT

Written informed consent was obtained from all study participants.

## Data Availability

The data in this manuscript are based on the scientific process and are completely true.
